# Supplementary material for: Magnetised quark nuggets in the atmosphere
Source: Sci Rep. 2021 Nov 17;11:22432. doi: 10.1038/s41598-021-01658-9 (PMC8599476; doi:10.1038/s41598-021-01658-9)
Supplement: Supplementary file 1 — Supplementary Information. [file 41598_2021_1658_MOESM1_ESM.pdf]

Supplemental Information for

Magnetised quark nuggets in the atmosphere

T. Sloan, J. Pace VanDevender, Tracianne B. Neilsen, Robert L. Baskin, Gabriel Fronk, Criss Swaim, Rinat Zakirov, and Haydn Jones

Supplemental Information on Methods: Sensor locations, topology and sound speed of the Great Salt Lake (GSL) Observatory for MQNs

The three sensors were anchored ~ 300 m apart on the points of an equilateral triangle centred on latitude 40.748683929^0, and longitude:112.287551880^0 in the southwest corner the Great Salt Lake, Utah, USA. The effective area for detecting MQN impacts is 6.4 x 10^7 m^2.

GSL contains a concentrated brine, not seawater. Sound speed was found to vary with depth and location, as the salinity and corresponding mass density of the brine varies as a function of depth. The sound speed was measured with a factory calibrated time of flight sensor. All measurements were in the water column, not the sediment. The resulting sound speed and standard deviation of the mean of the measurements versus depth are shown in Fig. S1 for Latitudes < 40^0 55.22′.

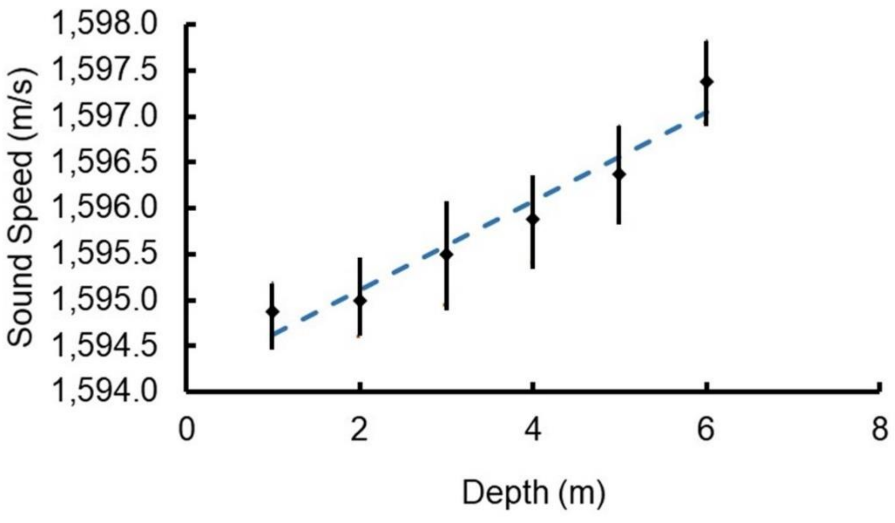

Figure S1. Data (black) and linear fit (dashed blue line) of sound speed vs. depth in GSL with scale on the left. Error bars are +/- 1 standard deviation of the mean.

The gradient in the sound speed c_s in Fig. S1 is $\Delta c_s/\Delta z \sim 0.5\ s^{-1}$. From Snell's law of refraction, a constant gradient curves a sound wave into a circle with radius R_s given by

$$R_s = c_s\left(\frac{\Delta c_s}{\Delta z}\right)^{-1} \approx 3200 \text{ m.} \tag{S1}$$

For a vertical explosion or MQN impact in water 5 m deep, sound waves originating at depth 0 to 5 m respectively reflect from the surface at radial distances of 0 to 180 m. Therefore, propagation has to consider refraction and attenuation in the realistic environment of the Great Salt Lake.

Supplemental Information on Methods: Explosive calibrations emulating MQN impacts

Explosive calibrations were conducted with 130-kJ or 260-kJ line explosives placed vertically in the ~5-m deep water column along lines to the north and east of the sensors. Depth versus distance for the two sets of calibrations is shown in Fig. S2.

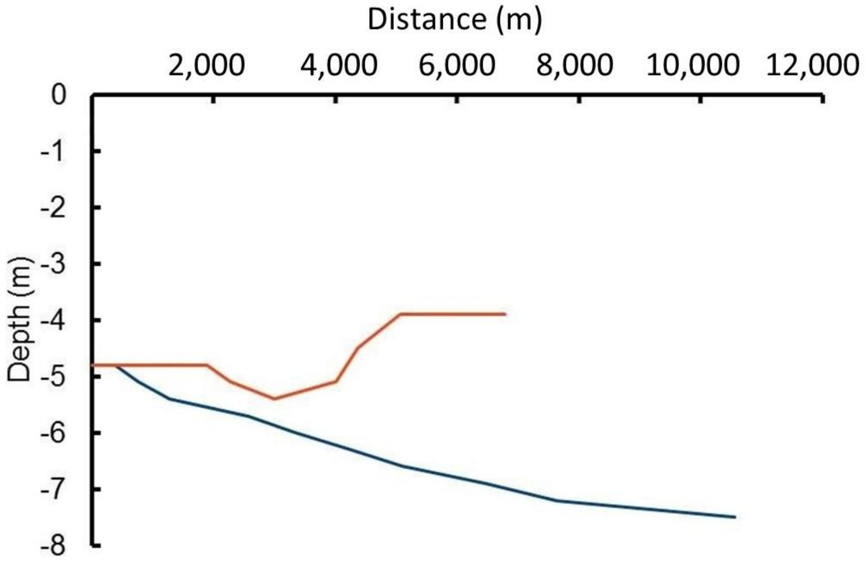

Figure S2. Depth versus distance is shown for the calibrations to the north (blue) and east (red) of the sensor.

PETN explosive cords of radius 2mm and mass 21.6 gm/m were used for the calibration shots. Single cords gave a deposited energy of 130 kJ/m and 2 cord shots 260 kJ/m. The high ~750 m/s detonation velocity of the PETN explosive gave a top-to-bottom asynchrony of only 0.7 ms, which is much less than the ~ 60-ms evolution of explosively produced channel, so PETN explosions are a reasonable emulation of an MQN impact.

Fig. 2 in the main text and Fig. S3 show samples of the calibration shots. These produced ~ 100 ms duration pulses with a distinct structure. The absolute value of the pressure has a precursor of frequency ~ 250 Hz with duration varying with distance from the hydrophone array, followed by ~ 20 ms burst with higher frequencies and higher amplitudes decaying to ambient conditions in another ~ 20 ms. The variation of the duration of the precursor burst with distance showed that it had group velocity 2.7% greater than that of the high frequency burst.

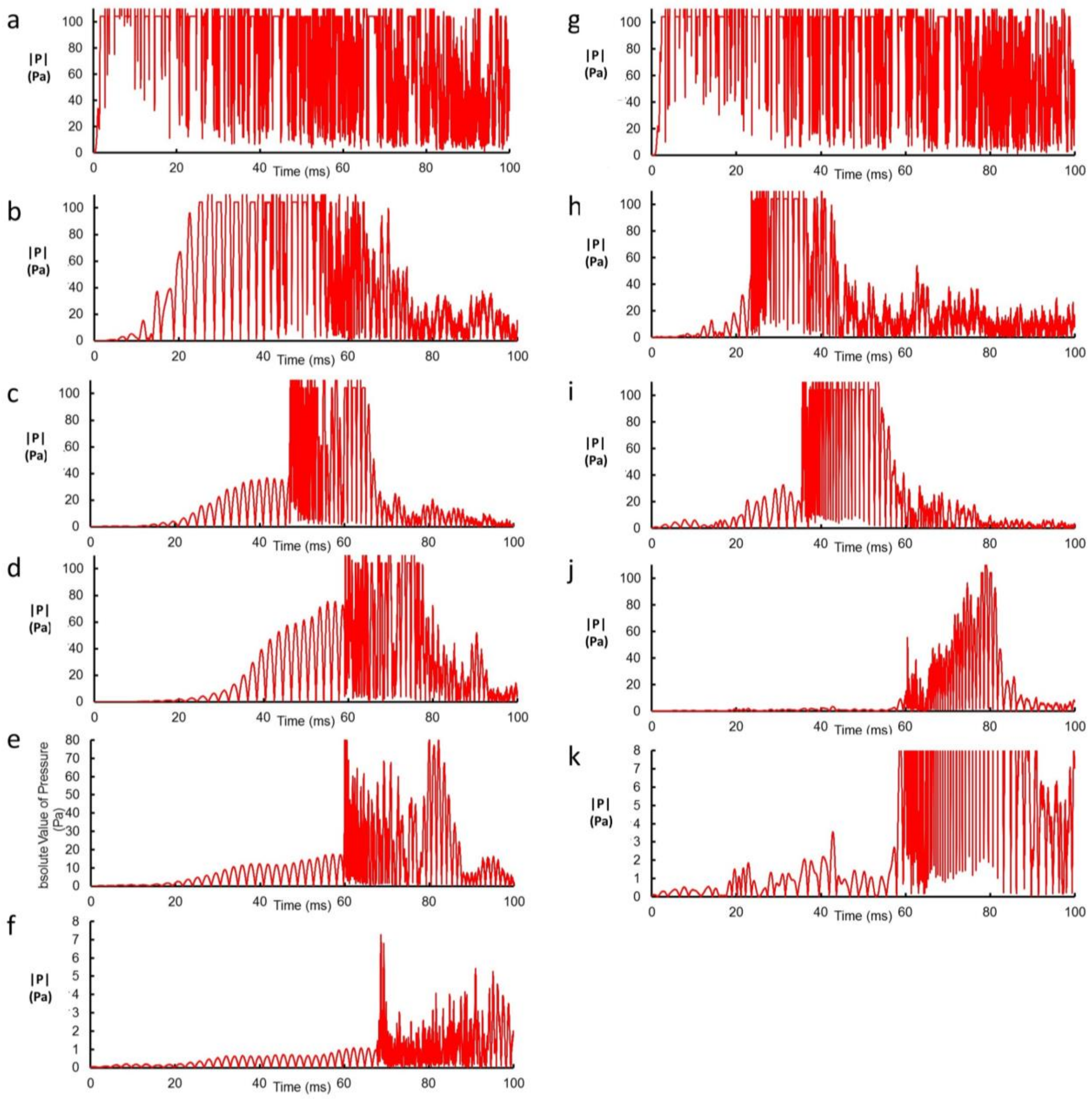

Figure S3. Absolute value of pressure in Pascals versus time in milliseconds at a2 hydrophone from the calibration shots. The explosive calibrations along the line east of the sensor (a through f from shots 5 through 10 respectively and at respective distances 150 m, 1230 m, 2210 m, 2180 m, 3100 m, and 4100 m) in approximately constant depth, as shown in Fig. S2, show a more regular oscillatory behaviour than those along the varying depth direction to the north (g through k from shots 1 through 4 at respective distances 150 m, 1050 m, 2060 m, and 2930 m). Line charges for d, e, and f had 260 kJ/m energy/length; all others had 130 kJ/m. Fig. S3 c and d provide a comparison of signals at the same position but with 130 kJ/m and 260 kJ/m respectively. All line charges were 4.8 m long. Fig. S3 k shows Fig. S3 j at an expanded scale to show the first 60 ms of pulse more clearly.

The absolute value of the pressure versus time for the nearly constant frequency portion of calibrations at approximately constant depth are shown in Fig. S4.

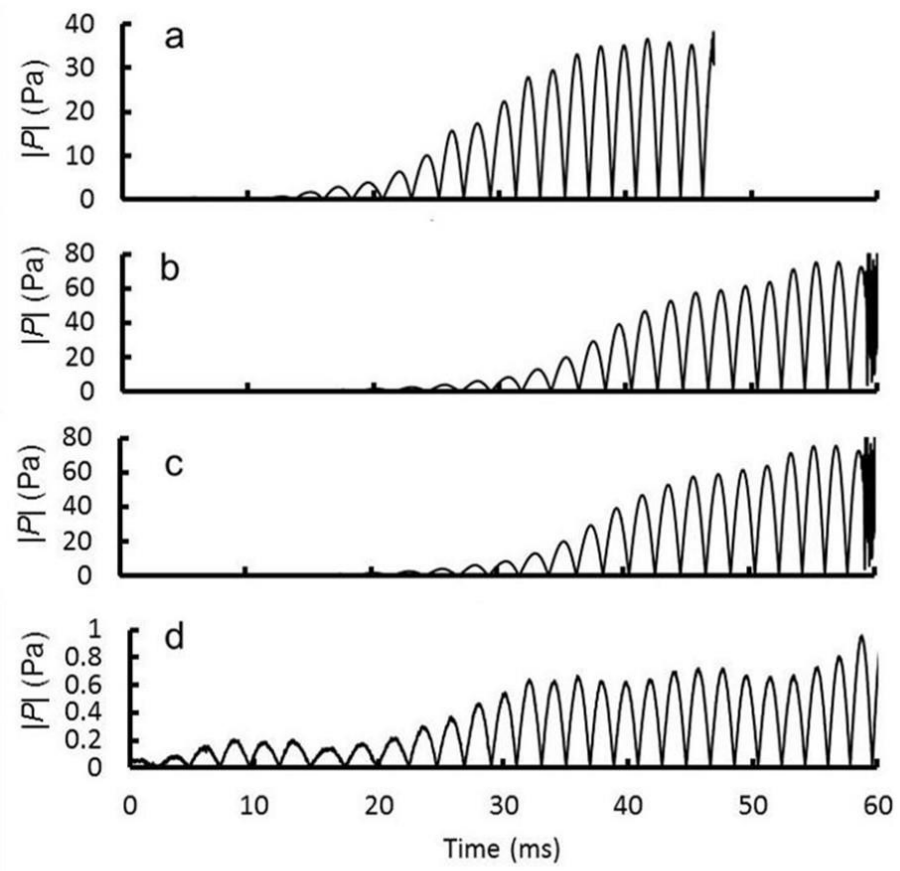

Figure S4. Absolute value of the pressure versus time prior to the arrival of the high-frequency pulse for explosive calibrations along a line to the east of the sensor. Distance and explosive energy in 5.0 m long cord follow: a) 2210 m with 130 KJ, b) 2280 m with 260 KJ, c) 3100 m with 260 KJ, and d) 4100 m with 260 KJ. The purity of the frequency content is evident.

The corresponding data from the explosive calibrations along a line to the north of the sensor with depth increasing with distance from the sensor are given in Fig. S5.

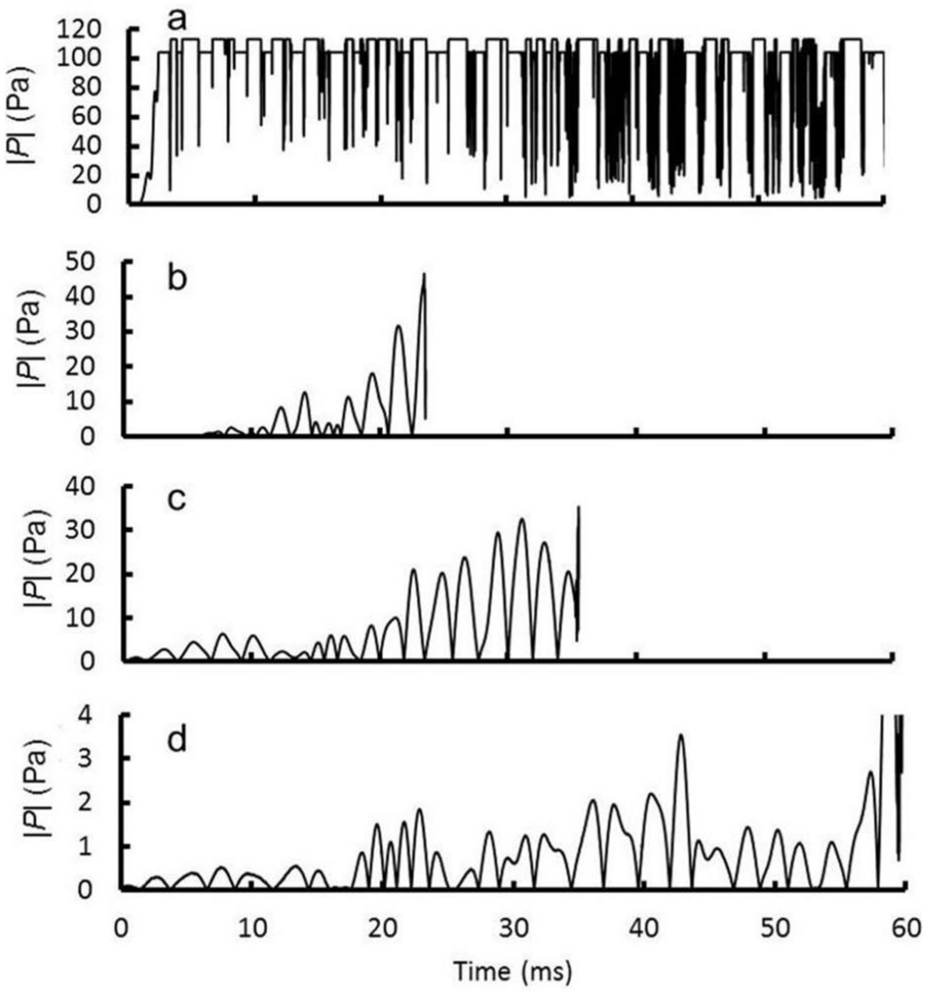

Figure S5. Absolute value of the pressure versus time prior to the arrival of the high-frequency pulse for explosive calibrations along a line to the north of the sensor. Distance and explosive energy in 5.0 m long cord follow: a) 150 m with 130 KJ, b) 1050 m with 130 KJ, c) 2060 m with 130 KJ, and d) 2930 m with 130 KJ. The frequency content of the signal is more complicated in this direction. The difference is attributed to the varying depth supporting multiple modes in the Pekeris waveguide.

Modelling using the ORCA Simulation in the Pekeris waveguide of the GSL.

The ORCA simulation proved to be very sensitive to the different assumptions made about the geology under the floor of the GSL, which is not well known and is described in the main text. The simulation for an ideal constant 5 m depth lake of brine produced conditions which matched observations when the sub-floor was assumed to have a hard top of thickness 0.75m over a 3m thick layer of mud with a mirabilite layer sitting on 5 m of bedrock. Fig. S6 shows the ratio of the resonant group velocity at 250 Hz to that at high frequency as a of mirabilite thickness. The arrow at mirabilite thickness 1.8 m shows the group velocity ratio of 1.03, the value obtained

from the calibration shots. The attenuation at this point was 30 db/km in reasonable agreement with the values obtained from the calibration shot data.

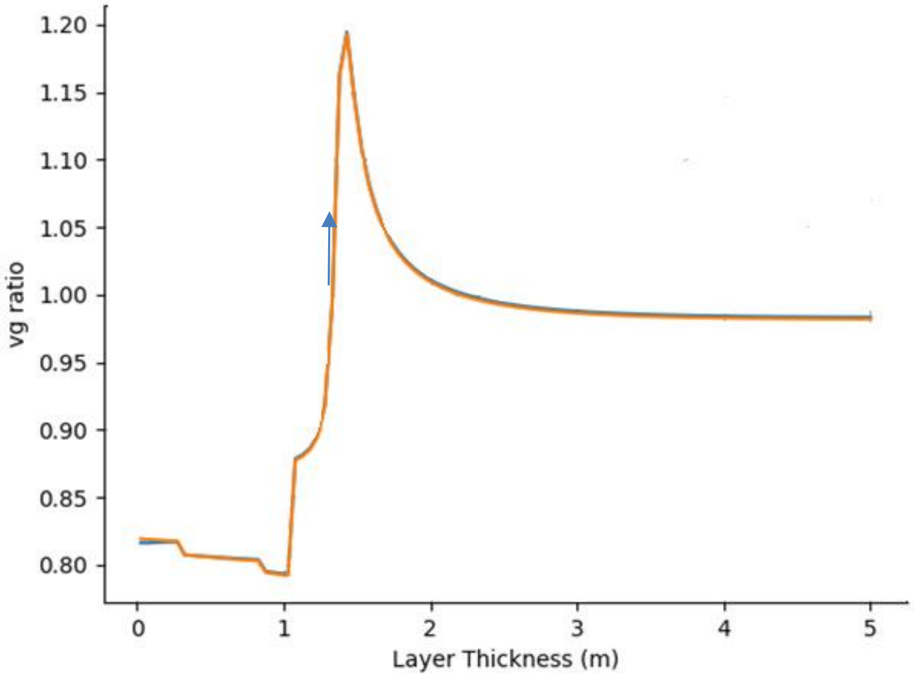

Figure S6. The variation with mirabilite thickness of the ratio of the group velocities of the resonant signal at 250 Hz to that at higher frequencies of 500 Hz (blue) and 1000 Hz (red). The arrow shows the mirabilite thickness that best fits the data.

Supplemental Information on Methods: Variation in trigger level from weather effects

The ambient background was surveyed every hour and the trigger level was adjusted accordingly, as discussed in the main text and is summarized in Table S1.

P_trigger (Pa)	Percent of Observation Time that signal with P > P_trigger can be recorded	Total Observation Time for signals with pressure above P_trigger (s)
0.2	20.00%	1.95E+06
2.5	80.00%	7.80E+06
5	86.00%	8.39E+06
10	89.00%	8.68E+06
20	90.00%	8.78E+06

Table S1. The variation of trigger level and recording time by trigger level.
